# Medical and Philosophical Intelligence

**Published:** 1817-11

**Authors:** 


					MEDICAL and PHILOSOPHICAL INTELLIGENCE.
THE receipt of the following has embarrassed us greatly. Whilst
it is painful to interfere with a contemporary Journal, it appears
not less illiberal and even cruel to refuse a foreigner that privilege
with which an Englishman is often indulged. We are besides under
obligations to M. von Embden for many valuable communications,
which more than entitle him to the space his defence will occupy.
We think it right to remark, that we have not taken the liberty we
often assume with communications from abroad in our own lan-
jio. 225. 3 l guage.
426 Medical and Philosophical Intelligence.
guage. What follows is printed exactly as we received it. We
shall conclude with a fervent wish, for our own sake and the benefit
of the public, that these contests may realize the adage of Hesiod
which immediately precedes M. Von Etnbden's quotation!
"LriKoy oe te ?ymova. ytiTU*
atyzvov ffftivSotn'. AyotSy *)i5s |3goroi<r?.
Dr. Von Embden in Reply to the Reviewer of the
Continental Medical Repertory.
If ever the sentence "dat veniam corvis vexat censura columbas"
Was in its proper place, 1 think I may apply it to myself. Aware
of the advantages to be derived from liberal criticism, I besought
the critics to let me reap the fruits of it, thus enabling me to im-
prove my work for the general benefit of the faculty. This appli-
cation, however, having been taken up erroneously, I was doubly
disappointed, for, instead of receiving the healing dew of the bee,
I obtained the poisonous sting of the wasp?inslead of meeting
with instruction founded on the basis of science and liberality, as I
had solicited and had a right to expect, I met with nought but
abuses resulting from rancour and acrimony. If information is not
the purpose of censorial remarks; if instruction is not to be gleaned
from them; if, instead of shewing us where we have erred, and
modestly directing us where we have failed, they only wish to
gratify their spleen, and let us feel their sarcasm, then indeed it
fcamlot be taken in good part, for then criticism misses its object,
and becomes debased to cavilling, and, as the poet says,
" Teach us to mourn our nature, not to mend
1 A sharp accuser, but a helpless friend!"
Unless the words of Hesiod be true, xoteh, I am
at a loss to conceive what could prompt the reviewer to indite me
of such grammatical ignorance as to be unable to write a single
line of English, or to impute to me such errors as no candid reader
would hesitate a moment to lay to the charge of the composer and
reviser. I have long enough had the honour of conversing with the
faculty of Great Britain to know how far I may confide in my
capability of conveying my ideas to them in their own language;
and too long have I been in the habit of holding in the highest
veneration the acknowledged liberality of the English public to-
wards foreigners, not to despise the effusions of the gall-dipped pen
of an illiberal individual, effusions which none but the hapless re-
viewer could write!?I lay no claim to any competency in the
English language beyond perspicuity, and well am I aware that it is
jiot in the power of every body, and, least of all, in that of myself,
to deliver my thoughts with elegance and in a flourishing style, yet,
as a judicious critic observes in one of our favourite journals,
*' Perspicuity is all that is required for the communication of prac-
tical points/' I am ceitain to have aimed at that qualification, and
dare likewise flatter myself to have attained it in a sufficient degree
to
Medical and Philosophical Intelligence. 4,27
to have been intelligible in every part of my writing, both in my
numerous communications in almost all the medical periodical pub-
lications in England and Scotland, and in the Continental Medical
Repertory. That I have been so in the latter, I am the more
fully persuaded, partly by the quotations the above-mentioned
respectable journal has done me the honour of taking from it, and
partly by the satisfaction it gave me to see, that, meagre in matter
and manner as the reviewer thought it, its matter was, nevertheless,
judged sufficiently rich to supply materials for nearly a sheet in his
own journal, and its manner such as not to let him hesitate to adopt
the systematical arrangement of the third department, into the
selectionary division of that very number in which he so candidly
delivered his opinion upon it!?Sic agitus censura!
That, however, the Continental Medical Repertory would be
more generally read in England, if written in good French than in
broken English, as the reviewer liberally expresses his opinion, j
cannot help doubting, as the major part of the faculty, particularly
its junior branches, would certainly find it more troublesome read-
ing in a foreign language than in their native tongue. Yet, for these,
I must own it might be translated among the selections, and even
save some editors the mortification of being under the necessity
of giving whole passages from Morgagni, to fill up their pages with
extracts from a work that has been printed and reprinted ever so
many times, both in original and translation!?Though, on the
other hand, were I inclined to return the compliment, I might be
led to conclude, from the misspelled and erroneous French quota-
tions we meet with in that Journal, that the Reviewer himself is
none of those that might be termed judges of the French language.
That, however, I do not wish to defend myself where l am cul-
pable, but, upon the principles of true disinterestedness, wish tQ.
mend whatever can be mended, as also that I shall be really thank-
ful for any instruction towards the actual improvement of my
Journal, will plainly be seen from the second number, which has,
been procrastinated on that very account, hut which will infallibly
appear on the last of the present month, and I hope it will convince
the faculty that neither expence nor pains will be spared to render
the Continental Medical Repertory worthy of their patronage.
Closing this my reply, I must beg the Editor's pardon for thus
piuch encroaching upon their valuable pages, assuring them that for
the future 1 shall take 110 notice of any continuation of the sarcasm
complained of in this, but that to this i found myself bound both
on account of myself and of the public. ?
Hamburgh;. Sept. 15, IS17. >
Extract of a Letter from Mr. Curtis to the Editor of thq
London Medical and Physical Journal.
SIR,
I beg leave to make some observations on a critique, which ap-
peared in your last Number, on my work on the Physiology and
Piseases of the Ear. That critique bears the strongest evidence
3 12 that
428 Medical and Philosophical Intelligence.
that it is not the production of any of the Editors of your Journal,
as the same, you must be conscious, appeared some time ago both
in the Morning Chronicle and Morning Post, being only in your
work lengthened out a little by quotations. As I consider it, there-
fore, the effusion of spleen, to which I should have thought you
would have been above giving countenance, you cannot, 1 trust, ob-
ject, as a matter of impartiality, to insert this in your next Number.
I believe you will admit, that no work can be entirely original,
and particularly when it is written for popular use; and that it is
the duty of the author to avail himself of all the previous know-
ledge on the subject. I have been much blamed, in this critique,
for incorporating in my work, along with my own views of disease,
the observations of the late Mr. Saunders; but I certainly should
have been wanting in respect to the merit of that eminent sur-
geon, had I either passed him over in silence, or treated his opinion
lightly. So much do I esteem his mode of practice, that it has
been my object to tread in his steps so far as he has gone, and to
endeavour to extend the views of the subject he had began: in
doing this, you, sir, for one, ought not to blame me, when the world
is so sensible how eager you have been to point out the merits of the
late Mr. Hunter, and to follow up his opinions and practice: this,
which if a credit to you, cannot be regarded as a discredit to me.
I am, Sir,
2, Soho-square; Your obedient Servant,
October 8th, 1817. John Harrison Curtis,
The remainder of Mr. Curtis's letter contains too much per-
sonal and extraneous matter for the London Medical and Physical
Journal. We submit it, therefore, to Mr. C.'s prudence to publish
it in any other chanuel he may think proper. We can assure our
readers, however, that the whole critique was by one of the customary
writers in our Analysis, and that he had never seen the " obser-
vations" in the morning papers to which Mr. C. alludes. It is true,
an anonymous critique was sent us, whether by a friend or an
enemy of Mr. C.'s we know not; but, as soon as we found it un-
favourable to the work, we determined, without further perusal,
not to avail ourselves of it,?a practice we constantly adopt on all
such occasions. If Mr. C. has not preserved a copy of his letter,
the original, or an attested copy, shall be sent to him.
It can scarcely be necessary to remind our readers of the dif-
ference between pointing out the merits of an author, and para-
phrazing his writings in such a manner as to make them appear our
own.?Edit.
To the Editors of the London Medical and Physical Journal?,
GENTLEMEN,
It is painful to record an instance of death from the imprudent
administration of colchicum, in a case of rheumatism, occurring in
one of our London hospitals; too large a dose had been giveq,
through the carelessness of the nurse, which produced constant vo-
piiting and purging, with agonizing pain in the stomach and bowels,
fo?
Medical and Philosophical Intelligence. 429
for the space of twenty-four hours?until the patient was relieved
from his sufferings by death. I cannot too often caution the pub-
lic against the use of this insidious and potent drug; and feel the
more regret, as I was the first to make known its powers in the re-
moval of the paroxysm of gout: though the readers of the Medical
Journal must ever bear in mind, that ! have stated, in the most dis-
tinct terms, that it was a medicine not to be trusted in the hands of
the public. The tinctures sold by Wilson, Reynolds, and Hyden,
are prepared according to my prescription for making the French
medicine,* and possess the power of relieving the pain of gout, but,
sooner or later, bring innumerable evils to the credulous patient.
1], North Crescent, Bedford-square. J. Want.
Extract of a Letter from Mr. Ward.?The antisyphilitic pro-
perties of the nitric acid, is a question which has excited much atten-
tion in the medical world. I beg leave to ofl'er a few observations
which occurred to me during my attendance at a military hospital,
where the nitric acid was administered with such assiduity as may
lead us to hope it is a medicine of some value after a proper use of
mercury. I am led to believe very good effects have been produced
by it in cases of syphilitic ulcers, particularly those of the throat and
in confirmed lues, where the patient's constitution has been melted
down as it were by the injudicious practice of introducing mercury in
large quantities into the system, with an idea of hastening the cure.
Practitioners well know, that in some such cases the use of mercury only
aggravates the disease and increases the malady. A gentleman of re-
pute in the medical profession relates a case in which the nitric acid
cured secondary symptoms of syphilis after the patient had undergone
two severe mercurial courses.f The nitric acid, when given to patients
in this stage of syphilis has produced very material benefit. I have
seen the secondary symptoms very soon relieved, the ulcers of the
throat have almost suddenly disappeared, and the constitution
greatly improved even in three weeks' time.? May not the proper-
ties of nitrous acid, when taken into the stomach, assimulate with
the juice therein; and, being taken up by the lacteals, form a kind
of mercurial preparation, by which means a fresh excitement in the
constitution may be effected, so as to ameliorate the disease, and in
some cases perfect a cure? To this action, I attribute the good ef-
fects I have frequently seen in syphilitic complaints of the worst
nature where it has been administered, and on which I shall shortlj
avail myself of an opportunity of a further communication.
Sloane-street.
On the 18th of October, being St. Luke's day, the College of
Physicians had their annual dinner and Oration- The orator,
Dr. Gibbs, of Bath, came to town on the occasion. The advan-
* We presume the same as prescribed in a former Number of our
Journal.?Edit.
t Vide Mr. Carmichael's paper, Medical and Physical Journal,
ypl. xxxiv. p. 464.
4 iajje
4S0 Medical and Philosophical Intelligence.
tage of a good voice and slow articulation gave a peculiar interest
to all he uttered, as there were few present who lost a single sen-
tence. The Oration was on the usual topics, but much fewer of
the defunct were mentioned, and of those few only the more illus-
trious dwelt upon; viz. Linacre, Hervey, Sydenham, and a few
others. To make out the number, Dr. Gibbs politely introduced
the elder living physicians of the city in which he resides. In the
early part of the Oration, the fanciful or empirico-regular practice
of medicine was noticed with much spirit. The devotees to mer-
cury, whether by friction or in blue pills, were pointedly and sar-
castically introduced; and the acid-baths did not escape.
The Oration was well attended; but nothing can exceed the
sombre and melancholy aspect of the Theatre, v. hich we fear there
is now no prospect of removing. We are informed that the College
%vas offered ground for a new building among the improvements
opposite Carlton-house; but, as the tenure would have been only
leasehold, it was, of course, declined.
An event, somewhat novel in the healing art, has attracted con-
siderable notice at the west end of the town. During the few last
liours of a celebrated Irish orator and lawyer, when his physician
(one of the Prince Regent's) was at the door to pay his customary
visit, he was informed that another doctor had been consulted by
one of the family, much interested in the life of the patient, and
who had a high opinion of this second doctor. The first being a
fellow of the College, as well as a royal physician, considered it
improper to meet one not regularly licensed, and expressed himself
somewhat strongly on the occasion. Unfortunately this was in the
presence of the party, who, without announcing himself, begged to
know the particular objection entertained against him. The answer
was most pointedly severe against the presumed irregular and his
supposed indecent writings. He now announced his name, and
begged a further explanation, which was refused. All that we
know of the matter since is by a copper-plate placardin which
the fellow of the College is not only accused of want of courage,
but of ignorance also. Application was made to the police officers,
but the supposed culprit has not hitherto been discovered.
Satisfactory as the Rev. Mr. Glover's account of Miss M'Evoy
must be to all impartial readers, it cannot be amiss to add, that it
has been confirmed to us by a conversation with another reverend
gentleman, long and well known for his philosophic acquirements,
the steadiness of his judgment, and his correct method of conducting
experiments. At the same time we feel it our duty to add, that the
number of sceptics even in Liverpool is considerable; and that we
liave heard reports which, till we can trace their origin, we do not
think ourselves called upon to publish.
* No letter-press printer chose to put his name to the placard,
nor, of course, ventured to print it without.
Curious
Medical and Philosophical Intelligence, 431
Curious Effect of Paste on Iron.?At Deanston, near the village
of Down, in the county of Perth, there is a manufactory where cot-
ton is woven by machinery. Iron cylinders were used in order to
apply the weaver's dressing to the cloth. This dressing, as is well
known, is nothing but common paste made of wheat-flour or barley-
meal. The cast-iron cylinder was in a short tii^e rendered quite
soft, and similar to plumbago, by the action of the paste. This
corrosion took place repeatedly; and it was so rapid that the pro-
prietors of the manufactory were obliged to substitute wood in
place of the iron. I conceive that the paste employed was usually
sour, and that it was the acid developed, which, by dissolving the
iron, produced this curious effect, A similar effect is produced
upon cast-iron by the action of muriate of magnesia, and probably
other salts.?Thomson's Annals of Philosophy.
On the Hedgehog.?The strong prejudices which are in many
parts of this kingdom entertained against that harmless animal the
hedgehog, or urchin, and the erroneous information respecting it
which has in some particulars been given by naturalists, induce me
to offer you the following observations, which are the result of near
two years' acquaintance with its habits.
The hedgehog subsists entirely on snails, slugs, worms, millipedes,
and other insects, and is consequently the best assistant the horticul-
turist can have in clearing his plants from those destructive vermin.
It never eats fruit, as it has been asserted by most zoologists that it
does; nor, as far as I have been able to ascertain, does it make
roots or any vegetable substance a part of its food. It is too gentle
and timid to attack young hares, partridges, or pheasants, as the ig-
norant gamekeeper will assert, to justify his persecution of a defence-
less animal: and the vulgar idea that it will suck a cow is too absurd
to require refutation. If put into a garden, it will in the course of
two or three nights entirely clear it of slugs; but of course it can
only be confined within a walled one, and it will be necessary to
feed it after the first few days, as it will not find a sufficient supply
of insects for its support. For this purpose a little raw meat, or
entrails, should be placed near its nest every other night; and it will
also require a little water in a shallow pan. It will secrete itself by
day in the most retired spot it can find, making its nest partly in the
ground, and covering it over with leaves. If disturbed, it generally
forsakes the place, and forms another habitation, whence it rarely
stirs but in the night. As far as I can judge, the confinement of
even a tolerably sized garden does not agree with the constitution
of the hedgehog, as I have generally found it prove fatal to him in
the course of five or six months. It would consequently be ad-
visable to keep him only for a limited time, then restore him to li-
berty, and have his place supplied by a fresh one. In winter it re-
mains in a torpid state, seldom coming from his nest unless the wea-
ther is very mild. It is, therefore, of little use to detain him from
his wild haunts, except in spring and summer.? Annals of Philos.
On preparing Extracts; by Mr. Johnson.?In the preparation
of
4.j3 Medical and Philosophical Intelligence.
of vegetable extracts, in evaporating diabetic urine, &c. the last por-
tion of water is expelled with most difficulty, and the last stage of
the process seems most injurious to the product. If small quantities
of rectified spirit be added occasionally, this stage is shortened, and
less injury is sustained.
The French chemists have, among other traits of their attention to
economics, been laudably industrious in turning to the best account
that prolific plant the potato ; and, during a late sojourn in
Paris, the writer collected the following particulars in regard to
two very important uses of its roots and its tops.
On the Distillation of Spirits of Wine (Alcohol) from Potatoes.
?A French lady, the Countess de N***, whom political events
compelled to change her chateau, on the banks of the Saone, for a
cottage eight leagues from Vienna?has established, on the small
farm she occupies, a distillation of brandy from potatoes; which
she has found to be very lucrative. The brandy of twenty degrees
of Reaumur is very pure, and has neither taste nor smell different
from that produced by the distillation of grapes. The method she
employs is very simple, and within every person's reach.
Take lOOlbs. of potatoes, well washed, dress them by steam, and
let them be bruised to powder with a roller, &c. In the mean time,
take 4lbs. of ground malt, steep it in luke-warm water, and then
pour it into the fermenting back, and pour on it twelve quarts of
boiling water; this water is stirred about, and the bruised pota-
toes thrown in, and well stirred about with wooden rakes, till every
part of the potatoes is well saturated with the liquor.
Immediately six or eight ounces of yeast is to be mixed with
twenty-eight gallons of water, of a proper warmth to make the
whole mass of the temperature of from twelve to fifteen degrees of
Reaumur; there is to be added half-a-pint to a pint of good
brandy.
The fermenting back must be placed in a room to be kept, by
means of a stove, at a temperature of fifteen to eighteen degrees of
Reaumur. The mixture must be left to remain at rest.
The back must be large enough to suffer the mass to rise seven or
eight inches, without running over. If, notwithstanding this pre-
caution, it does so, a little must be taken out, and returned when it
falls a little; the back is then covered again, and the fermentation is
suffered to finish without touching it?which takes place generally in
five or six days. This is known by its being perceived that the li-
quid is quite clear, and the potatoes fallen to the bottom of the
back. The fluid is decanted, and the potatoes pressed dry.
The distillation is by vapour, with a wooden or copper still, on
the plan of Count Ilumford. The product of the first distillation is
low wines.
When the fermentation has been favourable, from every lOOlbs.
of potatoes, six quarts and upwards of good brandy, of twenty de-
grees of the areometer, are obtained; which, put into new casks,
and
Medical and Philosophical Intelligence. 433
and aftenvards browned with burnt sugar, like the French brandies,
is not to be distinguished from them.
The Countess de N. has dressed and distilled per diem lOOOlbs.
of potatoes at twice, which gives sixty to seventy quarts of good
brandy. We may judge from this essay what would be the advan-
tages of such an operation, if carried on on a grand scale, and
throughout the year.
The residue of the distillation is used as food for the stock of her
farm; which consists of thirty-four horned cattle, sixty pigs, and
sixty sheep; they all are excessively fond of it when mixed with
water, and the cows yield abundance of milk. The sheep use about
five quarts per diem each; viz. one half in the morning, and one
half at night. The malt must be fresh ground. The Countess has
it ground every week.
On the Means of Extracting Potass from Potato-tops.?-One of
the most important discoveries of the present day is that of a drug-
gist of Amiens, by which Europe will be freed from the heavy tri-
bute she pays to America for the article of potass. The author of
this discovery has, in a truly patriotic manner, made known his dis-
covery?after ascertaining, by a series of experiments, the truth of
his conclusions. The French Society of Agriculture, and the So-
ciety for Encouragement of National Industry, have both handed
commissioners to frame official reports.
The following is an extract from the Neivry Telegraph of the
27th ult.?" A labourer in the neighbourhood of Newry, was
seized with the typhus fever, when in the employment of the Editor
of this paper. Medical aid having been procured for him, he was
ordered to the hospital, whither he was immediately sent. The
principal symptoms of his disease were a very rapid pulse, a burn-
ing skin, parched throat, and aching head. The man lay for a
short time in the hospital; when, seized by some kind of sudden
phrenzy, he sprang from his bed, and effected his escape, with no
other covering upon him than his shirt. In this state of almost
total nudity, he ran tjirough the town and across the field to his
own cabin. All efforts to induce him to return to the hospital
proved vain. On the night after this extraordinary exploit, he took
?ight grains of calomel, which in a few hours were succeeded by a
dose of Glaubers salts. In twenty-four hours more, the same me-
dicines were repeated with gpod effect. But 011 the fourth day of
the disease, the patient, the heat of whose skin was far above the
proper temperament, rose of his own accord from his bed, and
plunged into an old neglected fish-pond belonging to Mark Devlin,
Esq. where he effectually cooled himself by sporting and gambling
about like an otter, amidst weeds, water, and mud. The fever
immediately left him, and in a few days he was perfectly able to
follow his usual occupation. The cure of this man is to be clearly
attributed to the air-bath, the water-bath, and the calomel."
no. 225. 3 k. Remark%
434 Medical and Philosophical Intelligence.
Remarks on Worms found in the Human Body, their Origin, %c.
by Dr. J. J. Virey.
The human species, as well as other animals, domestic ones in
particular, are subject to nourish in their intestines and other organs
several species of worms, on which we shall present a few re-
flexions. It has appeared, in fact, so difficult to many naturalists,
as Goeze, Leclerc, Bloch, Pallas, &c. to explain their formation, that
several have thought it necessary to recur to spontaneous generation.
Rudolphii, whose treatise on Intestinal Worms is the most complete
(Carol. Osmund Rudolphi Entozoorum sive Vtrmium Intestinalium,
Histor. Natur. Paris, Amstel, &c. 1810, 3 vols. Svo. plates), counts
about fifteen species of worms which attack man.
1. Filaria medinensis, Gm. Rudolph, which is found in the
cellular tissue between the muscles: it has sometimes been seen in
the eye. This worm rarely attacks persons except within the
tropics.
2. Hemularia subcompressa, Treutler. This species is found
in the conglobate and lymphatic glands, and in the bronchial rami-
fications of the lungs.
3. Trichocephalus dispar. Rud. Discovered first by Morgagni.
Ascaris trichurca, Lin. Mastigodes, Zeder. Found in the large
intestines; also in monkeys.
4. Ascaris lumbrico'ides, Lin. previously described by Tyson,
Redi, Valisnieri, &c. Found in the small intestines; also in the ox,
the horse, the ass, and the pig.
5. Ascaris vermicularis, Lin. Keeps near the rectum and colon;
passes sometimes into the genitals externally.
6. Distoma hepaticum, Abilgaard and Rud. Fasciola hepa-
tica, Lin. Muller, and Blqch. Found jn the gall-bladder, from
whence it passes also into the intestines.
7. Poly stoma pinguicolq, Zeder. Hexathyrdium p. Treut-
XER. Yellowish worm, tjiree-quarters of an inch long, found ill
the ovarium of women.
8. Polystoma venarum, Zeder. Hexathyr, Treutler. Found
in the tibial vein on bleeding the foot: this worm is doubtful, and
imperfectly described.
9. Taenia solium, Lin., Audry, Carlish. Pretty common
in the small intestines, where it sometimes grows to 30 or 40 yards
long.
10. Tcenia lata. Lin., Pallas, Bloch, &c. &c. Very com-
jnon in northern climates.
11. Cysticercus cellulosat, Rud. previously described by Mai-
pigbi as found in pigs; confirmed by Hartmann and Fabricius.
Ab agea peudenti. Found in man between the muscles; sometimes
in the brain, as in sheep, and in the different viscera; several kinds
of monkeys are subject to it, but the pig particularly, which is at-
tacked by the hydatisjirma of Blume?bach.
12. Echinoccocus hominis, Ri^d. Polycephalus h. Goeze, &c.
Jound by Mickel in the liver and other viscera.
13. Diceras rude ditrachyceras rudis, Saltser. From the in-
4 * testipes^
Medical and Philosophical Intelligence. 435
testines, enveloped in a loose membrane, with two hard horns on
its head.
14. Strongylus giganteus, Rud. Found in the region of the
loins, and the bladder.
15. The crinons, common in horses, rare in children, coming
out of the back or breast. The comedones of the ancients have
been compared to the filariapapillosa, Rud.; but Chabert thinks
them a species of strongylus.
Report published by the Russian Counsellor of State, Lewshein",
(author of several Works on Rural Economy,) on a new Remedy
for the Hydrophobia.
There lived in the village of Sorokoletowo, in the circle of
Belewsky, government of Tula, an old soldier, who, I was told, had
frequently cured men and animals who had been bitten by mad
dogs. Having got some information on the subject, I learnt that
lie reduced into a powder a root similar to an onion; and that,
after having strewed it on a slice of bread and butter, he gave it to
the patients to eat, and 1 was assured that they were always cured
by it. I gave little credit to it, until an accident furnished me with
a proof of its efficacy. One of my brother's hounds went mad, and
bit the huntsman: the ordinary operation was performed to prevent
the propagation of the virus, the wouud healed, and we had no
uneasiness on the subject; but in a few weeks all the symptoms of
hydrophobia appeared, and we were obliged to confine the huntsman
with great precaution. As there was no medical man in the neigh-
bourhood, I advised the patient to be taken to the soldier* He
administered two doses of his remedy, one in the evening, the other
the next morning; and then said that the man might be unbound and
taken home without any danger. The huntsman experienced great
weakness, but he had no fits either of delirium or hydrophobia. In
a few days he found himself perfectly cured, and he has now lived
eighteen years without having a relapse. The soldier said that he
learnt the remedy of a peasant of Archangel.
The alisma plantago, or water plantain, is the one which this
man made use of. It grows in water marshes, lakes, and stagnant
muddy pools: the root resembles an onion, with thick fibres. This
plant remains under water till the latter end of May or the begin-
ning of June; when in flower it has a head like asparagus. It is in
flower all the summer, and may be gathered at any time; but the
best is at the end of August. The roots are well washed and cleaned,
and dried in the shade; when dry, it is pulverised, and administered
as above. Two or three doses have always been found sufficient to
effect a cure, even after the hydrophobia is declared in the patient,
whether it be men or animals, that have been bitten by mad dogs,
in the cure of which even it has scarcely ever been known to fail;
Indeed, during the twenty-five years that it has constantly been
practised in the government of Tula, no instance of failure in an
immense number of cases, has been known,
Sip,
436 Medical and Philosophical Intelligence.
Sir James Earle was favoured with an euthanasia suited to
the close of a tranquil life. Descended from an ancient and wealthy
family, of which he was a younger son, he had all the advantages
of a learned education, when, at a suitable age, he was apprenticed
to Mr. Pott,?at that time the most celebrated surgeon in Europe.
Soon after the expiration of his apprenticeship, he was chosen as-
sistant-surgeon to the principal hospital in the metropolis, married
his master's daughter, and, before he was too much advanced in
life to avail himself of such a distinction, became a senior surgeon
to the same establishment. His publication of his father-in-law's
works, and his improvement in the operation for the Hydrocele,
are too well known to require further mention.
He was so sensible of his approaching end as to send to one of
his brethren of ihe College, in order to determine on the necessary
arrangements concerning his oftice as Master, and his consequent
duty as public orator for the Hunterian oration. Both these he
was advised to resign, which he cheerfully did, and expired before
the completion of the succeeding twenty-four hours.
Dr. Berlioz has lately published a volume on the Good Effects
Of Evacuations [sanguineous] and Acupuncture in Chronic Diseases,
from which we shall exlract some remarks relative to the latter re-
medy. Acupuncture, as practised among the Chinese, consists in
the introduction of a sharp needle of gold or silver to a certain depth
on the part affected, where it is allowed to remain for four or five
minutes. It is either pushed in slowly with the fingers, or driven, in
quickly by a small mallet. M. Berlioz prefers the former method.
This author relates several cases where acupuncture has been ser-
viceable; among others, is that of a young woman who had long
been afflicted with a slow nervous fever, accompanied by obstinate
pain in the stomach. A needle was pushed in at the pit of the sto-
mach, so as to penetrate that organ. " L'epingle, dit-il, enforcee
deloute sa longeur dans l'epigastre, avoit certainement penetre
jusqu'il l'estomac." A man was instantaneously cured of a con-
vulsive cough by a similar process. According to Berlioz, the in-
troduction of several needles is not more efticacious than that of one.
? The good effects certainly do not proceed from counter-irritation,
since the introduction is frequently unaccompanied by any pain, and
is then most successful.
Dr. Johnson, author of the Influence of Tropical Climates on
European Constitutions, &c., has just completed a condensed trans-
lation of Guilbert's new Work on Gout, which is a transcript of the
celebrated article on that subject in the 19th volume of the Dic-
tionnaire des Scienccs Mcdicales, and forms a large Appendix to
Dr. Johnson's work lately announced, and which will appear on the
1st of December, under the following title?The Influence of the
Atmosphere, more especially the Atmosphere of the British Isles, on
the
Mr. Salisbury's Botanical Excursions for August. 437
the Health and Functions of the Human Frame; embracing Obser-
vations on the Nature, Treatment, and Prevention, of the principal
Diseases resulting from sudden Atmospherical Transitions; and un-
folding Original Views and Fundamental Principles for the Prolon-
gation of Life and Conservation of Health. To which are added,
Practical Researches on the Pathology, Treatment, and Prevention
of Gout and of Rheumatism, in all their Proteian Forms.
Extract of a Letter from a Correspondent in the North.
I wish to draw the public attention to a grievance I know not
"Well how to remedy. I have at present under my care three cases
of infectious fever, viz. typhus, all imported here (for the benefit of
their relations) in the public coaches!!?the worst of the three, under
night, in the mail, in company with three unsuspecting travellers.
Mr. Salisbury's Botanical Excursions for August and
September.
August ISth. Hyde-park and Bayswater.
Myriophyllum spicatum |
Chenopodium Bonus
Henricus
Euphorbia Paralais
Mentha rotundifolia
Elymus caninus
Antirrhinum spurium
Melissa Nepeta
Osmunda spicant
Erigeron canadense
Datura Stramonium
Senecio Sarrecenicus
Sium angustifolium
Angelica sylvestris
Dipsacus sylvestris
Peucedanum Silaus
Leonurus Cardiaca
Conium maculatum
Sagittaria sagittifolia
Seaum Telephrum
dasyphyllum
Pimpinella saxifraga
Origanum Onites
Allium Vineale
CentaureaCalcitrapa
Artemisia vulgaris
Poa aquatica
Phleum paniculatum
Sambucus Ebulus
Sempervivum Tectormn
Statice Armeria
Poterium Sanguisorba
Picris hieracioides
Campanula liapuncultis
Convolvulus ai vensis
Circcea lutetiana
Saponaria officinalis
Caucalis nodosa
infest a
Crepis biennis
Heracleum Sphondylium
Lycopsis arvensis
Phalaris arundinacea
August 25th. Battersea-fields and Wands worth-common.
JLysimachin tnyrsiflora
Lepidium latifolium
Lulium t'amulentum
Chironifi Centaurium
Cistus 'ileliantheniuin
Corian.drum sativum
Hyosciamus uiger
Vero,nica scuteliata
Cic'norium Intybus
La ctuca scariola
? . virosa
Convolvulus sepium
'Clinopodium vulgare
Erysimum cheitan-
thoides
Zannicliellia palustris
Geranium sylvaticum
Chenopodiuni olidum
ficifolium
urbicum
Retonica officinalis
Atropa Belladonna
Avena fatua
Gnaphalium margarati-
ceum
Althaea officinalis
Apium graveolens
Arctium Lappa
Briza media
Crepis tectorum
Anetlium Fajniculum
Galium Mollugo
Bromus hirsuius
- ? ? 1 ?- secalinus
Caucaiis daucoides
Dip^acus pilosus
Sisoii segetum
Stachys sylvatica
Scirpus acicularis
Clematis Vitalba
Erica cinerea
Ononis spinosa
Lotus corniculatus
Euphrasia officinalis
Myosotis scorpioides
Anthemis nobilis
Tormentilla reptans
Carduus acanthoides
LysimaehiaNummularia
Anthemis arvensis
ItaphanusUaphauistrum
September
458 Report of Diseases*
September 3d. Battersea-fields, and the neighbourhood.
Pteris Aquilina
Veronica scutellata
Origanum vulgare
Senecio Jaoboea
Stachys germariica
Scirpus acicularis
Inula dysenterica
Senecio sarrecenicus
Polygonum Fagopyrum
Leonurus Cardiaca
Melissa Nepeta
Antirrhinum minus
Centaurea Calcitiapa
Lysimachia vulgaris
Solidago Virga aurea
Ericu multiflora (gar-|
dens)
Dabcecia (ditto)
Arundo Calamagrostls-
Bidens Cernua
tripartita
Stachys palustris
?' - arvensis
Sagittaria sagittifolia
Panicum crus galli
viride
verticillatuta
September 17th. Gravesend and its neighbourhood.
Aster Tripolium
Clienopodium mariti-
nium
Atriplex pedunculata
Chiora perforata
Samolus Valerandi
Artemisia inaritima
Nepeta Catrvria
Arenaria peploides
Crocus officinalis
Erigeron canadense
Conyza squarrosa
Zosteria marina
Scabiosa succisa
A<;rostis littaralis
Valeriana rubra
Ophrys spiralis
Scilta autumnalis
Dipsacus prlosus
Chironia Centaurium
Salicornia repens
herbacea?
REPORT OF DISEASES.
At length the epidemic fever has been sufficient to attract the
public notice, and several physicians, Drs. Brine, Bateman, Adams,
Curry, and probably others, have offered their opinions in the daily
prints. If typhus, typhoid symptoms, contagion, and other words
so often used without a precise meaning, should now acquire each
its appropriate distinction, we may truly say, with our Flemish
correspondent, ayaQv yfa fipoloio-w.
From our own knowledge, we can assert that the fever; though
most common, and generally mild, in the recesses of poverty, is by
no means confined to them. And, though it rarely, if ever, spreads
in well-ventilated apartments, yet, when it attacks those who are
better fed, the symptoms are much more severe, and net to be re-
lieved without venisection or topical bleeding. If these? or other
evacuations, are disregarded in the beginning, the physician has
ample cause of repentance, and the patient of complaint, in the
protracted progress of the disease.
Some of the dysenteries, treated improperly in the beginning,
have become fatal; others are in that chronic, yet painful, state, as
to leave little doubt of ulcerations in the inner coat of the intestines,
and, consequently, to threaten a fatal issue.
The woman under phthiriasis has been much relieved by an in-
fusion of helebore, with which she sponges herself twice a-day; but
neither this nor all her attention to cleanliness is sufficient to keep
her free from these disgusting vermin.
Dr. Charles Parry will speedily publish^an Appendix to Dr.
Parry's Experimental Inquiry into the Nature of the Arterial Pulse,
&c. &c.; containing the result of certain experiments announced ia
that work.
439
A METEOROLOGICAL JOURNAL,
By Messrs. William Harris and Co. 50, Holborn, London,
From the 20th September to the 19th October, inclusive.
Rait)
gauge
Sep.
20
21
22 ,04
23
24
25 O
26 ,09
27
28 .10
29
30 ,06
Oct
1
2
3 C
4
5
6
7
8
9
10
11
12
13
14
15
16 .21
17 O -54
18 .09
19 -08
THERM.
5763,156
58 63 53
5558
59 63
Barom,
30
30
299
29?
29 9
29 6
292
294
29?
299
30
29?
30
301
301
301
302
301
301
30
299
299
30
302
302
30
299
30
29s
299
30
299
29
29
29
294
29
29
299
299
30
299
301
30*
301
30
30
30
30
29
299
30
301
30
301
299
29
30
299
299
De Luc's
Hygrom.
Dam i
Wind.
WSW
NE
NNE
NNE
NE
S
S
sw
sw
sw
NW
NE
N
NE
NNE
N
NNE
E var.
E
E
N
NNW
N
N
NE
NNW
N
N
NE
NE
W
NE
NNE
NNE
S
S
SW
WSW
w
sw
NE
NW
NW
NNE
NE
NE
NNE
E var.
E
E var.
N
NW
N
NNE
N
N
NE
NE
NE
ENE
Atmospheric
Variation.
Fine
CIo.
Fine
CIo.
CIo.
Rail
Rain
Fine
Fine
CIo.
CIo.
Fine
Fine
Fine
Fine
Fine
Fine
Fine
Fine
Fine
Fine
Fine
Fine
Fine
Foe.
CIo.
Rain
Rain
Rain
Rain
The quantity of rain fallen in the month of September ia
61-100ths of an inch only.
LECTURES

				

